# New Approach to Privacy-Preserving Clinical Decision Support Systems for HIV Treatment

**DOI:** 10.1007/s10916-022-01851-x

**Published:** 2022-10-20

**Authors:** Gabriele Spini, Emiliano Mancini, Thomas Attema, Mark Abspoel, Jan de Gier, Serge Fehr, Thijs Veugen, Maran van Heesch, Daniël Worm, Andrea De Luca, Ronald Cramer, Peter M.A. Sloot

**Affiliations:** 1 Applied Cryptography and Quantum Algorithms, TNO, 96800, 2509 JE Postbus, The Hague, The Netherlands; 2 Institute for Advanced Study, University of Amsterdam, Oude Turfmarkt 147, 1012 GC Amsterdam, The Netherlands; 3 Cryptology Group, CWI, P.O. Box 94079, 1090 GB Amsterdam, The Netherlands; 4 Mathematical Institute, Leiden University, P.O. Box 9512, 2300 RA Leiden, The Netherlands; 5 Philips Research, High Tech Campus 34, 5656 AE Eindhoven, The Netherlands; 6 Complexity Institute, Nanyang Technological University, Academic Building North, Level 1 Section B Unit No. 7 (ABN-01B-07), 61 Nanyang Drive, 637335 Singapore, Singapore; 7 Advanced Computing, ITMO University, Lomonosova street 9, 191002 Saint Petersburg, Russia; 8 Department of Medical Biotechnologies, University of Siena and Siena University Hospital, Viale Mario Bracci 16, 53100 Siena, Italy; 9 Department of Global Health, Amsterdam UMC, Location AMC, 1105 AZ Amsterdam, The Netherlands; 10 Data Science Institute, Hasselt University, Diepenbeek, Belgium

**Keywords:** Clinical decision support systems, Anti-HIV agents, Secure multiparty computation, Privacy, Confidentiality

## Abstract

**Background:**

HIV treatment prescription is a complex process. Clinical decision support systems (CDSS) are a category of health information technologies that can assist clinicians to choose optimal treatments based on clinical trials and expert knowledge. The usability of some CDSSs for HIV treatment would be significantly improved by using the knowledge obtained by treating other patients. This knowledge, however, is mainly contained in patient records, whose usage is restricted due to privacy and confidentiality constraints.

**Methods:**

A treatment effectiveness measure, containing valuable information for HIV treatment prescription, was defined and a method to extract this measure from patient records was developed. This method uses an advanced cryptographic technology, known as secure Multiparty Computation (henceforth referred to as MPC), to preserve the privacy of the patient records and the confidentiality of the clinicians’ decisions.

**Findings:**

Our solution enables to compute an effectiveness measure of an HIV treatment, the average time-to-treatment-failure, while preserving privacy. Experimental results show that our solution, although at proof-of-concept stage, has good efficiency and provides a result to a query within 24 min for a dataset of realistic size.

**Interpretation:**

This paper presents a novel and efficient approach HIV clinical decision support systems, that harnesses the potential and insights acquired from treatment data, while preserving the privacy of patient records and the confidentiality of clinician decisions.

## Background and Significance

The constantly rising cost of national healthcare [1] associated to an aging population has highlighted the need for a critical change in traditional healthcare [2, 3]. Most stakeholders (clinicians, healthcare providers, policy makers and patients) agree that the solution lies in new approaches in which technology and health information technology (HIT) play a critical role [4, 5]. HIT services aim to automate and optimize healthcare processes with the overall goal of providing a more effective treatment process for patients. One of the main barriers to the adoption of HIT lies in the challenges associated with the need to preserve the privacy of the patients’ data; legislation on the privacy of sensitive data, such as the General Data Protection Regulation (EU) 2016/679 (GDPR), is becoming increasingly more stringent, affecting all parties who handle sensitive data.

In this paper, we focus on one specific category of HIT systems: *Clinical Decision Support Systems* (CDSSs). A CDSS is a system that provides clinicians, patients, and other individuals with intelligently processed disease-specific and patient-specific data. Several different categories of CDSSs can be found in literature, such as diagnostic tools, expert systems, and workflow support. Systematic reviews reported that CDSSs significantly improved clinical practice: a review [6] on one hundred studies reported improvements for more than 62% of the trials on practitioner performance, reminder systems, drug-dosing systems and disease management systems. A review on seventy studies [7] reported a significant improvement of clinical practice in 68% of trials. Recent systematic reviews [8, 9] report an improvement in health care processes in 148 randomized, controlled trials and in 85% of twenty-two studies respectively.

As a use case to present our proposed solution to the problem of preserving the privacy of patients’ data, we focus on an expert system for HIV treatment. The prescription of antiretroviral drugs to HIV1 infected patients is a complex process in which clinicians have to take into account several factors in a short amount of time. In particular, clinicians need to choose the most appropriate treatment based on the genotype of each patient’s most prevalent strain of the virus in order to minimize drug resistance. A suboptimal treatment will likely result in a more rapid emergence of drug-resistant strains, and, eventually, in increased morbidity and mortality.

CDSSs are used in order to minimize or, ideally, prevent the prescription of suboptimal HIV1 treatments. Some examples of relevant CDSSs range from simple quality improvement consultation programs like HIVQUAL-US [10] that monitors clinical performance, to more sophisticated data-driven systems like Euresist [11] and knowledge-based systems like the HIVdb Program [12]. The main advantage of the use of these CDSSs is that they save a considerable amount of the clinician’s time, since it would be impossible for the clinicians to analyze in detail the differences between the HIV genotype that is prevalent in a specific patient in search of critical mutations. However, in this paper we focus on the “comparative Drug Ranking System” (cDRS), a CDSS that helps to minimize the choice of sub-optimal HIV treatments by performing a meta-ranking analysis of three expert systems for HIV-1 genotypic drug resistance interpretation (ANRS, HIVdb, Rega) to resolve possible discordances between them [13–16]. The discordances in drug resistance between the three expert systems are not negligible [17, 18], and are the result of the limited amount of clinical data available for each specific set of mutations and of different methodologies used by the systems. A CDSS able to help clinicians in resolving such discordances is essential to avoid the administration of sub-optimal HIV treatments.

Research on the spread of the HIV epidemics has led to the development of tools (such as phylogenetic trees) able to correlate specific viral sequences in different patients and reconstruct with good accuracy the network of infections within a community [19]. In addition, transmission events between patients can be identified by analyzing the viral genotypes, given the uniqueness of specific sets of mutations [20, 21]. Hence, strict privacy regulations prevent the sharing of patient data (e.g., viral genotype) that feed and improve these clinical decision support systems. Moreover, clinicians might not be able, or willing, to openly share their treatment decisions and the resulting outcomes, even though such information might be beneficial for the decision-making process of their colleagues. In conclusion, there is a tremendous amount of valuable information that is unavailable to clinicians because of privacy and confidentiality constraints.

An ideal system should allow clinicians to compare their chosen treatment against the outcome of the treatments chosen by other clinicians for similar genotypes, while solving the issue of utilizing patient and clinicians’ data in a secure, privacy-preserving way.

In this exploratory work, we present a solution that uses cryptographic techniques, namely a so-called *secure Multiparty Computation (MPC)* protocol, to achieve this functionality without violating any of the privacy constraints. Informally stated, MPC is a collection of cryptographic techniques that allow several parties, each of which holds some private input, to evaluate a function on those inputs without disclosing any extra information on the input themselves, and without resorting to a trusted external party. Our MPC-based solution would allow clinicians to compare past treatments of ‘similar’ patients to find the optimal treatment for new patients preventing any unauthorized party, including the ones performing the computations, to access the input data.

### Related Work

Privacy-preserving CDSSs have been presented in recent years [22–25]. However, this line of work focusses on CDSSs for disease-prediction and enables clinicians to securely query remote machine-learning based systems for a given patient’s health condition, in a privacy-preserving way. As such, it is not directly comparable with our solution, which has a different scope within the paradigm of privacy-preserving CDSSs.

In more general terms, proposed applications of MPC to the healthcare sector have flourished in recent years. To the best of our knowledge, there exists no article summarizing the scientific literature on MPC applied to the healthcare sector^1^; we provide here a list of recent and relevant work on the topic, but we stress the fact that this list cannot be exhaustive, due to the high number of publications on the topic.

A large sub-domain of the application of MPC (and other related cryptographic techniques) to the medical domain aims to deploy machine-learning techniques on medical datasets held by distinct organizations; examples in this sense include privacy-preserving reinforcement learning [26], Kaplan-Meier survival analysis and genome-wide association studies [27], grid logistic regression for biomedical data [28], training of linear [29] and Lasso [30] regression models on medical data, and computing patient risk-stratification metrics [31].

Other relevant work include medical record searching [32, 33], the study of general methods such as privacy-preserving data mining for joint data analysis between hospitals [34] and branching programs for privacy-preserving classification of medical ElectroCardioGram signals [35], the presentation of specific use case scenarios such as secure disclosure of patient data for disease surveillance [36], R-based healthcare statistics [37], and privacy-preserving genome-wide association study [38], privacy-preserving genome analysis [39] and search of similar patients in genomic data [40].

Finally, iDASH [41] is an important public initiative to stimulate the development of techniques for privacy-preserving sharing of medical data.

### Outline

The rest of the article is organized as follows. In the “Materials and Methods” section we first discuss how to measure the effectiveness of a treatment from patient records and present the method that we propose (setting aside the privacy-preserving aspect); we then provide a brief overview of MPC and of the framework of our choice, SPDZ. In the “Results” section we then explain how the effectiveness measure is securely implemented within SPDZ and present an evaluation of the efficiency of our solution. Finally, the “Discussion and Conclusions” section summarizes the results of the article and provides an appraisal of the achieved results and on possible future work.

## Materials and Methods

### Measuring Treatment Effectiveness from Patient Records

The viral genotype of a patient refers to the genetic sequence(s) of the HIV-1 virus strain that is most prevalent at the time of the blood test. The HIV-1 virus RNA genome contains 3 key regions that encode for enzymes critical to the life cycle of the virus: *protease (P), integrase (I)* and *reverse transcriptase (RT)*. Each region encodes for enzymes with 99, 288 and 560 amino acids, respectively, all of which could in principle mutate. These mutations play an important role in the drug resistance of the virus strains.

Given an HIV-1 patient, our goal is to obtain treatment results of ‘similar’ patients, and therefore we need to define a metric or distance function that quantifies the similarity between two patients, or two viral genotypes. Since all expert systems indicate resistance to drugs based on substitutions in the amino acid sequence of the wild-type HIV-1, we need a way to compute the distance in the amino acid sequences of the viral proteins. Metrics of distances between amino acid sequences are fairly complex and often assessed via neural networks [42]. The assignment of a suitable similarity metric is outside the scope of this paper, and for this reason, we have chosen to use a simplified viral genotype representation with a generic metric as a proof of concept. However, our solution is flexible, since it can support other representations and metrics.

From now on we shall represent viral genotypes as bit strings *v* of a fixed length *N*, i.e., $v\in {\left\{\text{0,1}\right\}}^{N}$. We can think of each bit in this bit-string as an indicator for the presence or the absence of a specific mutation at a specific position.

Since there are only 97 relevant positions with commonly 1 or 2 resistance-associated substitutions [64] we can expect $N$ to be somewhere between 100 and 200. The distance between two viral genotypes $v_{1}$ and $v_{2}$ is defined by the Hamming distance between the bit strings:

$$H\left({v}_{1},{v}_{2}\right)=\left|\left\{i : {v}_{1}\left(i\right)\ne {v}_{2}\left(i\right)\right\}\right|,$$

Given this metric we can define two viral genotypes *v*_1_ and *v*_2_ to be similar if their Hamming distance is smaller than a certain threshold *B*, i.e., $H\left({v}_{1},{v}_{2}\right)<B.$ Even though this metric is a simplification of the metrics used in practice, it is quite similar to the rule-based metrics used in the CDSSs of [13, 16]. These CDSSs match viral genotypes based on the presence of resistance-associated substitutions in amino acid positions, which can be seen as a Boolean expression. In a clinical setting, these CDSSs compare the two complete viral strings to identify specific insertion, deletions, and substitutions but do not rely on a single threshold value defined as a Hamming distance. In fact, it is well known from specific studies which additions, deletions or substitutions trigger a clinically relevant mutation. In a practical implementation, we would have to look at the difference in specific positions of the sequences of two amino acid strings. The threshold that would be used in that case would be defined by clinicians who set of rules used by the specific CDSS instead of the Hamming distance described in the example. However, the rulesets that would trigger an alert are still Boolean in nature and would fit the proposed secure MPC solution.

Suboptimal treatments of HIV-1 patients result in faster emergence of resistant strains and this emergence renders the treatment ineffective. Hence, a way to measure the effectiveness of a treatment *tr* for genotype *v* is by indicating the *time-to-treatment-failure* $TT{F}_{tr}\left(v\right)$. The $TT{F}_{tr}\left(v\right)$ is defined as the time (in days) between the start of a therapy *tr* and either a therapy switch, a discontinuation of therapy or death [65, 66], for a patient with genotype *v*. Hence, given an HIV-1 patient with genotype *v* we would, for example, like to compute the average $\overline{TTF}_{tr}\left(v\right)$ over all patients with similar genotype *v*_*i*_, as an indication for the unknown true effectiveness measure $TT{F}_{tr}\left(v\right)$:

$$\overline{TTF}_{tr}\left(v\right)= \frac{1}{\left|\left\{i : H\left(v,{v}_{i}\right)<B\right\}\right|}\sum _{i : H\left(v,{v}_{i}\right)<B}TT{F}_{tr}\left({v}_{i}\right) ,$$

Where *H* denotes, as discussed above, the Hamming distance and *B* denotes a fixed threshold value.

### Secure Multiparty Computation

MPC has been introduced by Yao in the 1980s [43]. Given *n* mutually distrusting parties ${P}_{1},\dots , {P}_{n}$, each holding private inputs *x*_1_,…, *x*_*n*_, the goal of MPC is to allow the parties to compute the value $f({x}_{1},\dots , {x}_{n})$ of a function $\text{f}$ on their inputs, without revealing any other information than $f({x}_{1},\dots , {x}_{n})$, and without resorting to an external trusted party.

Early research in the 1980s [43–46] established the theoretical feasibility bounds for MPC; informally stated, this line of research proved that any function *f* with finite domain and finite image can be evaluated securely in an MPC fashion. The precise security properties that can be achieved depend on the behavior of players and on the underlying communication model.

Since the first market-ready deployment of MPC in 2008 [47], MPC solutions have been used in various practical contexts, e.g., stock market order matching [48], job market inquiries [49], and frequency bands auctions [50]. Moreover, various software suites and implementation frameworks for MPC have been made available [51–55].

Several considerations have to be made when applying MPC to a given problem. For instance, one may assume that parties ${P}_{1},\dots , {P}_{n}$ will behave semi-honestly (meaning that they may try to learn information on the other parties’ inputs, but do follow the protocol), or that it is instead necessary to provide security against fully malicious players that deviate from the protocol instructions. Another important parameter that varies among protocols is the number *t* of corrupted parties that can be tolerated out of the total number *n* of parties.

A remark of notable importance is that many desirable properties of MPC may negatively impact performance, or even be mutually exclusive, which means that the choice of an MPC protocol may be subject to important trade-offs. The reader can refer to [56] for a comprehensive discussion of MPC.

### The MPC framework of our choice: SPDZ

We base our MPC solution on the SPDZ protocol [57, 58]. The protocol is distinguished for its fast performance, and is implemented in a freely accessible software suite called SPDZ-2 [52, 54] for UNIX-based systems^2^; SPDZ-2 allow developers to write programs in Python-like syntax, and it then compiles the code to executable format.

SPDZ follows the so-called *share-compute-reveal* paradigm: each input *x*_*i*_ of the function *f* to be computed is ‘dispersed’ (or, formally speaking, *secret-shared*^3^) into *n* pieces of data, called shares, each of which is assigned to a party; this process has the property that no information on *x*_*i*_ can be extracted from a set of shares, unless such a set contains *all* shares (in which case *x*_*i*_ can be completely recovered). Subsequently, parties execute a ‘computation’ protocol; as a result of this step, each party will have a share of the output $f\left({x}_{1},\dots ,{x}_{n}\right)$ of the function. Once all shares have been gathered, the output can then be reconstructed.

**Fig. 1:**
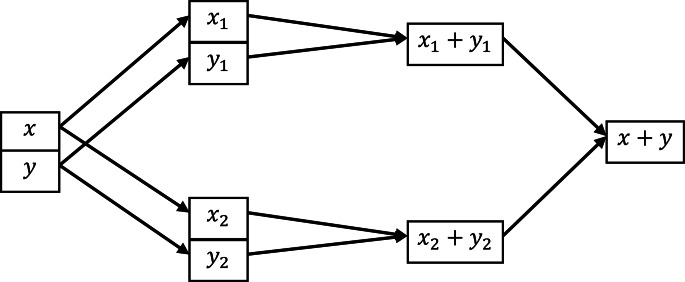
Example of share-compute-reveal paradigm for addition of two values

A schematic representation of this paradigm for the addition of two values *x* and *y* among two parties is provided in Fig. 1. The top row represents the shares held by the first party, while the bottom row represents the shares of the second party. The assumption here is that *x*_1_ and *x*_2_ are two random values subject to the condition that ${x}_{1}+{x}_{2}=x$, and similarly for *y*; bearing this fact in mind, it is then seen how the process respects the privacy and reconstruction requirements discussed above.

In more general terms, the core idea behind MPC protocols based on the share-compute-reveal paradigm is that the function $f$ to be evaluated on the input values is “decomposed” into basic operations (such as sum and products); these basic operations are then translated into similar operations on the shares and executed in the same order. An important remark is that, in general, these operations on shares require some form of interaction among the parties (for instance, multiplication of two values cannot simply be performed by multiplying the corresponding shares, and requires a more involved and interactive process). The reader can refer to the literature on MPC and on SPDZ that we have provided for a more formal and complete discussion of this topic.

Other cryptographic techniques such as homomorphic encryption [59, 60] could potentially be of relevance for private data analysis, but we ruled out these alternatives, because they would induce a huge computational overhead in our setting.

The share-compute-reveal approach is particularly well-suited for the client-server model we are interested in: the ‘input’ parties, clinicians (clients) simply need to supply their secret-shared inputs to two or more ‘computing’ parties (servers), who will execute the computation protocol on these inputs, and then communicate the shares of the output to the input parties, which can thus reconstruct the output.

It is important to remark that the SPDZ protocol does not, *per se*, distinguish between input and computing parties. A framework for MPC in a client-server model was presented in [61]; moreover, in [63] the SPDZ protocol was adjusted to the client-server setting.

The SPDZ protocol is divided into an ‘offline’ phase and an ‘online’ phase. The offline phase can be executed before the function inputs *x*_1_,…,*x*_*n*_ are known, and its goal is to produce some secret-shared auxiliary data that will be used in the evaluation of $f$; producing this data can be a computationally-intensive process, but since secret inputs are not required, this step can be executed during idle time and well before the actual secure computation will take place. Once the auxiliary data has been produced, the evaluation of $f$ can be performed very efficiently: this is of particular relevance for our use case, where input parties (clinicians) need to obtain the output of the function $f$ within a matter of minutes, while preprocessing material can be produced in the background by the computing parties.

## Results

The functionality we have achieved utilizes HIV patient records to gain new insights in the effectiveness of HIV treatments. The MPC protocol ensures privacy of the patients and the confidentiality of the clinicians’ treatment decisions.

**Fig. 2:**
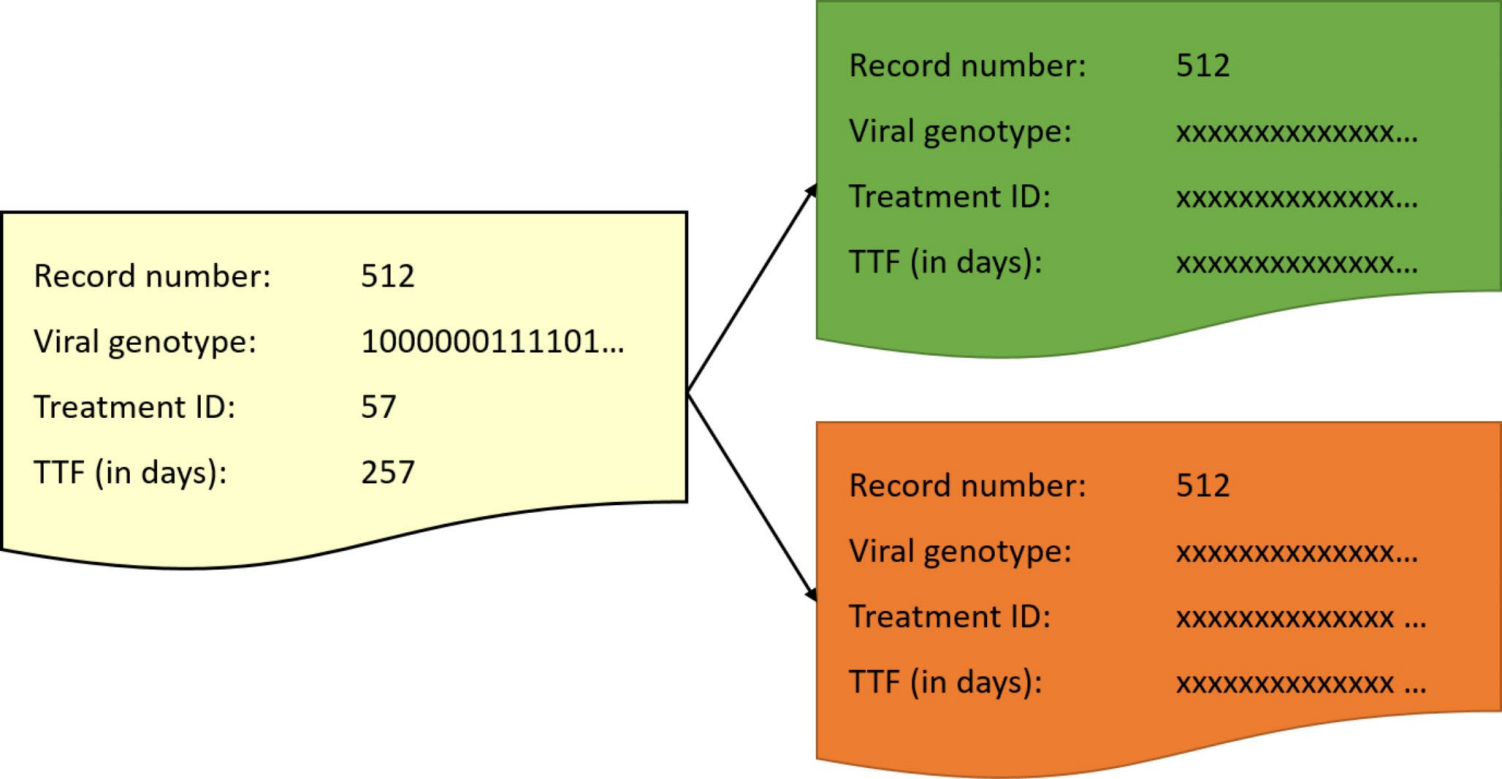
Secret sharing database records

The proposed solution distinguishes between ‘input’ parties, the clinicians supplying the database records, and ‘computing’ parties running the SPDZ protocol, which can be different medical institutions or IT service providers. The input parties additively secret-share their data records and distribute the shares amongst the computing parties (see Fig. 2).

**Fig. 3:**
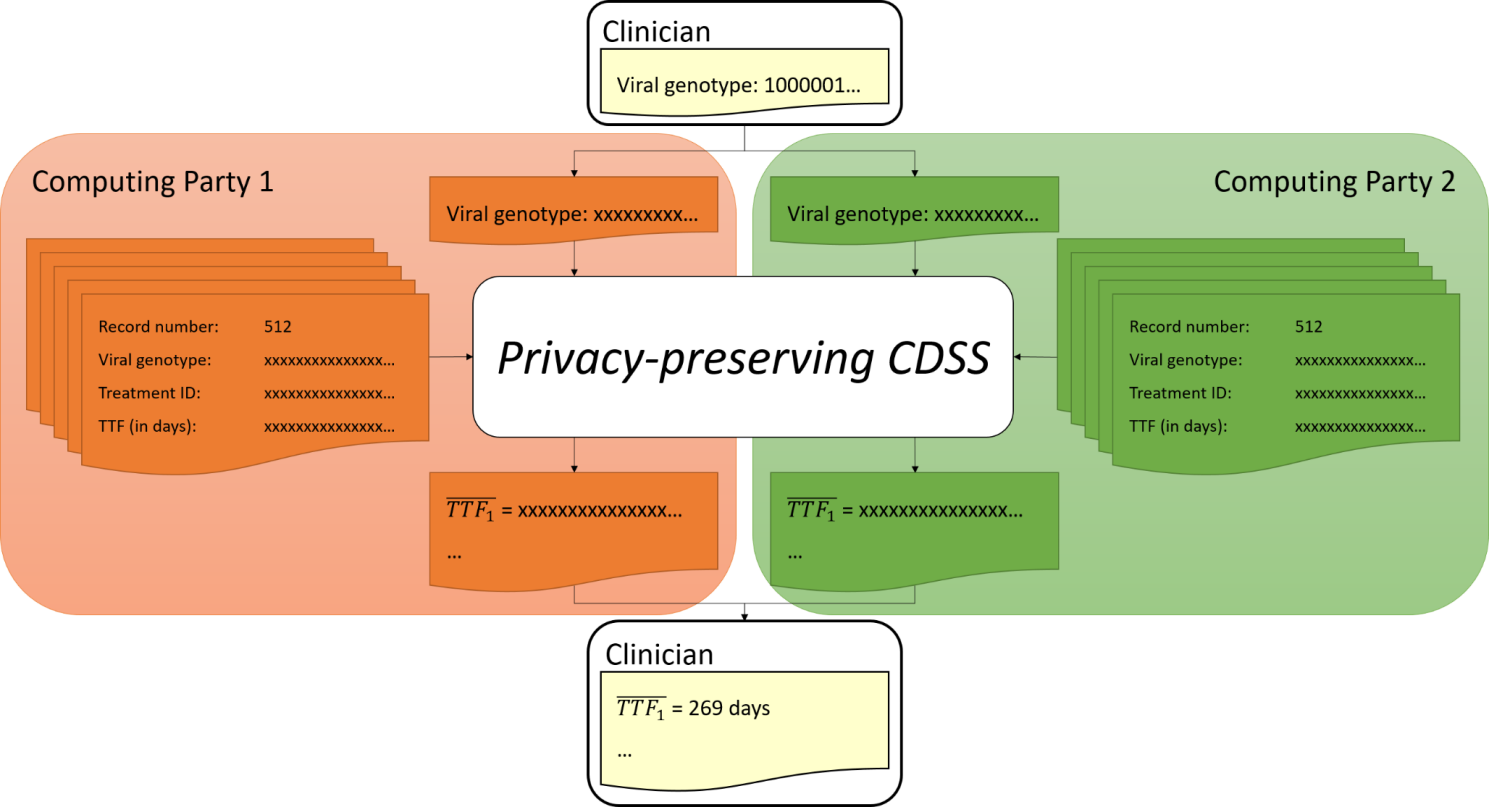
Query architecture of the privacy-preserving CDSS

As a result, the two computing parties each hold a share of all the database records. SPDZ allows the evaluation of queries to this secret-shared database in such a way that only the output of the query (the average time-to-treatment-failure ( $\overline{TTF}$) per treatment) is revealed to the clinician, and no additional information is leaked to either the querying clinician, or the computing parties (cf. previous section). In order to protect the private information in the query (the viral genotype), we secret-share the query amongst the computing parties in a similar manner. The computing parties thus take as private inputs their shares of the database records and their share of the query. They do not reconstruct the result of the computation (the average *TTF*) themselves; instead, each of them sends their share of the result to the querying clinician who, in turn, recombines the shares to reconstruct the output. This way the result is only revealed to the clinician, and not to the computing parties (cf. Figure 3).

Our solution allows clinicians to compare their treatment of choice against the outcome of treatments previously chosen by other clinicians for patients with similar genotype, without revealing any private information to the clinicians or the computing parties, who only learn the size and format of the database and the number of queries to the database. This system is secure as long as the two computing parties do not collude.

### Performance – Online Phase

In comparison to implementing the functionality without privacy protection, using MPC inherently introduces computational and communication overhead. The main reason for this unavoidable overhead is that, in an MPC protocol, the computation path has to be oblivious, i.e., independent, of the input values, since it would otherwise leak information. Moreover, as explained in the previous section, some basic operations on the input data are translated by MPC into more complex, interactive processes, which lead to unavoidable overhead.

We have evaluated the performance of the online phase of our protocol by deploying the computing parties on two different machines, each using one core of a i7-7567U CPU running at 3.50 GHz and 32 GB of RAM, in a local network with 1 Gbit/s throughput. The system ran on a Fedora operating system and has been developed within the SPDZ-2 software suite discussed in the previous section; the overall orchestration of the scalability experiments has been performed via scripts for the Bash shell. Finally, we have instantiated the SPDZ protocol with 40-bit statistical security, 128-bit computational security and a 128-bit prime field.

The experiments that we have run measure the time it takes for the solution to return the average time-to-treatment-failure $\overline{TTF}_{tr}\left(v\right)$ of a given input treatment *tr*, where the additional input value *v* is the genotype of a given patient. The formal definition of $\overline{TTF}_{tr}\left(v\right)$ is presented in the “Materials and Methods” section; as a reminder, it is given by the average over the times-to-treatment-failure of patients with a similar genotype, for the same treatment *tr*.

**Fig. 4:**
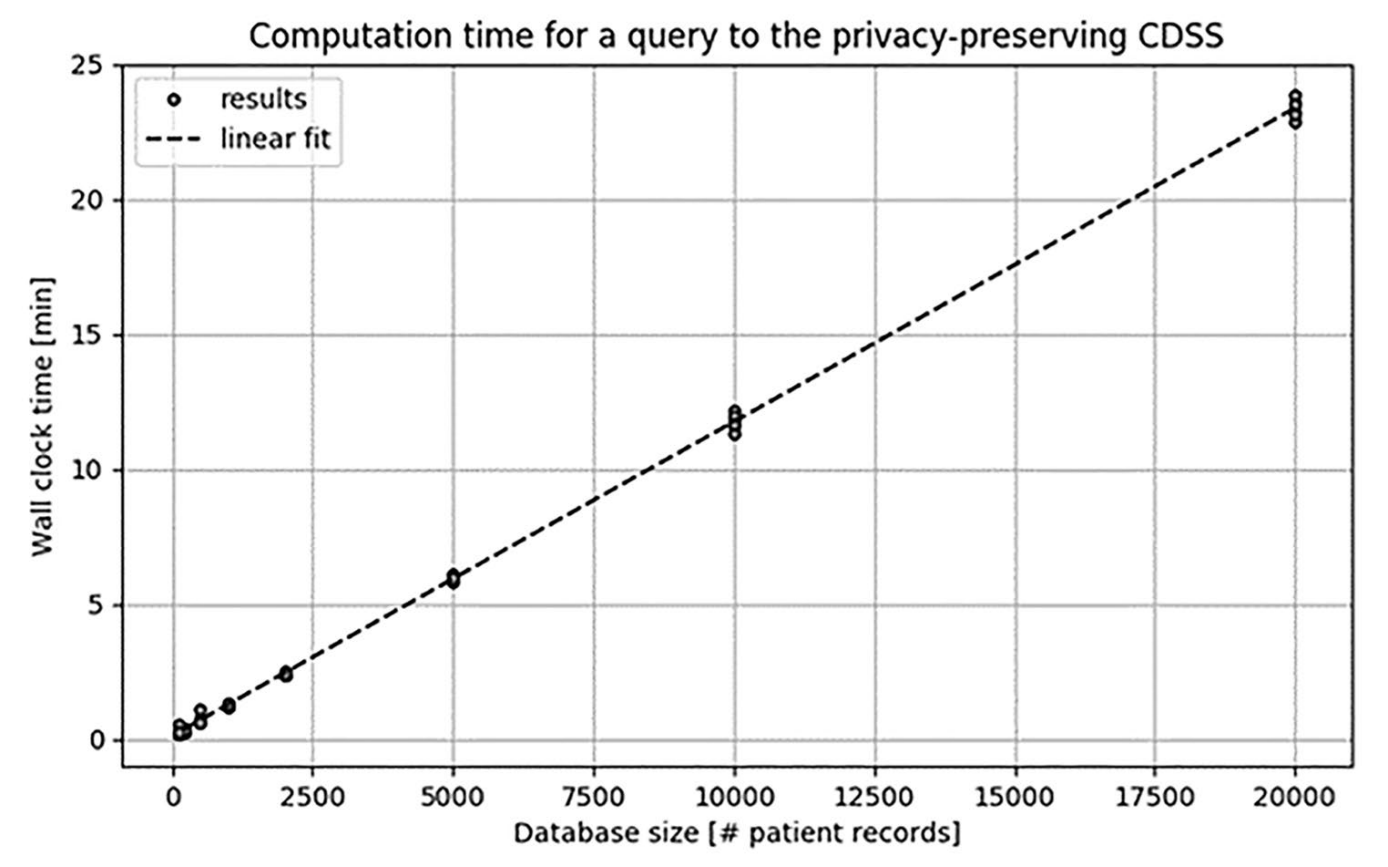
CDSS computation time

The results in Fig. 4 show the computation times that are needed for answering one query, for artificially-generated databases with sizes ranging from 100 to 20 000 records. The maximum 20 000 approximates the number of HIV-positive registered individuals in the Netherlands [62]. The experiment is repeated multiple times, resulting in several data points per database size. Recall that per query we compute the average *TTF* conditioned on ‘similar’ patients for 100 different treatments. The computational complexity scales linearly in the number of database records. Notice that the threshold value *B* and the number of patient genotypes that have Hamming distance at most *B* from the given input do not affect the running time of the computation: this is inherent to MPC solutions, which have a computation time which does not depend on the input values.

Also notice that these figures refer to the time needed to answer a single query; with the current state of our implementation, the running time would scale linearly in the number of queries. This is an aspect to be kept in mind should, for instance, a practitioner want to query the system for different values of the threshold *B*.

### Performance – Offline Phase

In the SPDZ protocol certain computational tasks are executed in the offline phase, that is independent of the MPC use case and that can be implemented with existing protocols. For this reason, we have merely estimated the computational costs of it. The offline phase can be run at any time to generate a large database of preprocessed data which, in turn, is consumed during the online phase.

The performance of the offline phase can be quantified in the number of the so-called multiplication triples that are generated per second. In [63] various approaches for generating multiplication triples in a setting similar to ours were evaluated, generating 30 000 triples/s. To evaluate a single query on a database with 20 000 records approximately 40 million multiplication triples are required. In this setting these triples can thus be generated in approximately 22 min.

## Discussion and Conclusions

We presented a novel approach for HIV1 clinical decision support systems, making use of advanced cryptographic techniques to process private information without revealing it. By making use of MPC, we can ensure both the privacy of the clinicians’ treatment choices and the privacy of patients.

Towards a fully operational deployment some points are yet to be addressed. Notably, the SPDZ software framework is designed for research purposes only, which means that our implementation should be audited and checked for vulnerabilities. For what concerns efficiency and scalability, we stress the fact that any CDSS for HIV treatment should produce a suggestion within minutes, since practitioners would typically query the system right after visiting a patient and would expect an answer before the patient leaves their office. As shown in Fig. 3, our solution answers a query within 24 min, for a database size roughly matching the number of HIV-positive registered individuals in the Netherlands [62]; while we consider this result to be sufficient for the proof-of-concept presented in this paper, some further work would be needed for a full-scale deployment. The running time of the implementation could be improved by several means, e.g., by using a low-level but very fast programming language such as C, by further parallelizing the computation, or by making use of high-performance computing machines instead of consumer-level hardware.

## Data Availability

No real data or material has been used for this article.

## Footnotes

1 One literature overview on privacy-preserving medical data sharing has been produced, but with a focus on blockchain-based applications.

2 Notice that support for the SPDZ-2 software suite (implementing the eponymous MPC protocol) has been discontinued. Development moved to forks SCALE-MAMBA and MP-SPDZ, both implementations of the SPDZ protocol.

3 It is important to notice that ‘sharing’, here, is by no means a synonym of ‘revealing’; on the contrary, it can be seen as a strong form of distributed *encryption.*

## Code Availability

The code produced in the work described in this article is currently not publicly available.
